# Low Blood Lead Levels Do Not Appear to Be Further Reduced by Dietary Supplements

**DOI:** 10.1289/ehp.8605

**Published:** 2006-04-18

**Authors:** Brian L. Gulson, Karen J. Mizon, Michael J. Korsch, Alan J. Taylor

**Affiliations:** 1 Graduate School of the Environment, Macquarie University, Sydney, New South Wales, Australia; 2 Commonwealth Scientific and Industrial Research Organisation, Exploration and Mining, North Ryde, New South Wales, Australia; 3 Department of Psychology, Macquarie University, Sydney, New South Wales, Australia

**Keywords:** blood lead, children, diet, female adults, micronutrients

## Abstract

**Objective:**

Our objective was to evaluate the association of dietary intakes of selected micronutrients and blood lead (PbB) concentrations in female adults and in children.

**Design:**

With longitudinal monitoring, we measured daily intakes of the micronutrients calcium, magnesium, sodium, potassium, barium, strontium, phosphorus, zinc, iron (limited data), and copper from 6-day duplicate diets (2–13 collections per individual) and PbB concentrations. Participants were three groups of females of child-bearing age (one cohort consisting of 21 pregnant subjects and 15 nonpregnant controls, a second cohort of nine pregnant migrants), and one group of 10 children 6–11 years of age.

**Results:**

Mean PbB concentrations were < 5 μg/dL. A mixed linear model that included only group and time accounted for 5.9% of the variance of the PbB measurements; neither the effect of time nor the effect of group was significant. The model containing all of the micronutrients (except iron, for which there was a great deal of missing data), along with time and group, accounted for approximately 9.2% of the variance of PbB; this increase was not statistically significant. There was, however, a significant association of PbB with phosphorus, magnesium, and copper when all micronutrients were included in the statistical analysis, perhaps reflecting a synergistic effect.

**Conclusions:**

In contrast to most previous studies, we found no statistically significant relationships between the PbB concentrations and micronutrient intake. In adults and older children with low PbB concentrations and minimal exposure to Pb, micronutrient supplementation is probably unnecessary.

Despite decreasing blood lead (PbB) levels, there are groups, usually disadvantaged, still at risk of lead exposure, such as children living in older, deteriorating housing and who have elevated PbB concentrations (Clark et al. 1985; Lanphear et al. 2002). Apart from primary prevention, such as safe removal of leaded paint, and removal of Pb from gasoline and Pb solder from canned foods, nutritional intervention is considered to play a critical role in reducing uptake of Pb (Mahaffey et al. 1974). Although dietary intakes replete in nutrients such as calcium, iron, zinc, and occasionally copper have been advanced as inhibitors of Pb uptake through the gastrointestinal tract, in many human studies only diet and blood samples were analyzed for Pb and other elements such as Ca, phosphorus, and Fe. Analysis of Fe is usually undertaken because of the association of anemia and elevated PbB levels in children (e.g., Bradman et al. 2001; Mahaffey et al. 1976; Markowitz 2000; Willows and Gray-Donald 2002). Furthermore, most of the information for PbB–micronutrient intakes comes from the 1970s and 1980s when intakes of Pb via diet and PbB values were orders of magnitude higher than now and for the subjects in our studies.

As part of a longitudinal study of mobilization of Pb from the maternal skeleton during pregnancy and lactation, we measured a suite of elements from 6-day duplicate diets collected every quarter. In addition to the usual elements of Ca, Fe, and Zn, we also analyzed samples for elements such as barium and strontium that are related chemically to Ca and may play important roles in bone remodeling. For example, although it has been recognized for decades that Sr plays a role in bone formation and/or resorption, a new drug Sr ranelate has been shown not only to decrease bone resorption but, in contrast to other bone resorptive drugs, also to build up bone mass (Reginster et al. 2005).

In this article, we have attempted to establish potential associations in mainly female adults between PbB levels and daily micronutrient intake and decide if certain of these micronutrients are beneficial in lowering PbB levels. The hypothesis is that there will be an inverse association between PbB level and micronutrient intake. In a previous article, we reported the progress results for dietary intakes for four of the five groups described here (Gulson et al. 2001).

## Materials and Methods

### Subjects

**Figure 1:**
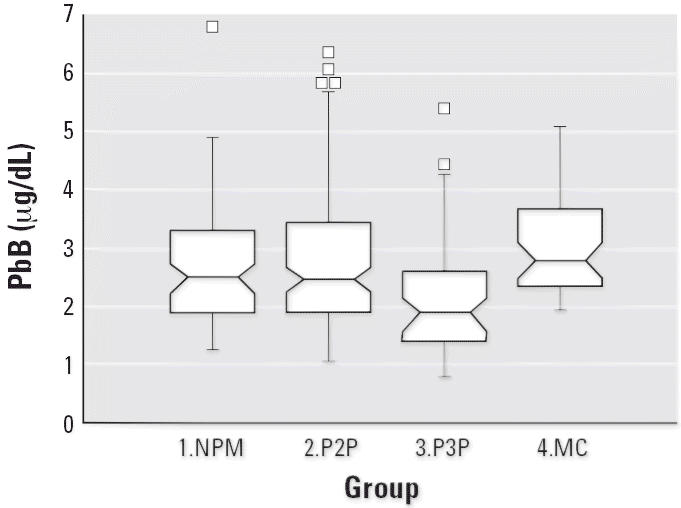
Notched box plot for descriptive statistical data of PbB levels in the four groups showing the overall low PbB levels. The result for a phase 2 subject whose PbB was 20 μg/dL on arrival in Australia is not plotted. In Figures 1–13, 1.NPM denotes group 1, nonpregnant migrants (*n* = 15); 2.P2P, group 2 (phase 2), pregnant subjects (migrant and Australian, *n* = 36); 3.P3P, group 3 (phase 3), pregnant subjects (*n* = 9); and 4.MC, group 4, migrant children (*n* = 10).

Our results are based on three groups of female adults currently living in Australia whose bone stores of Pb acquired between the ages of 0 and 35 years are from isotopically different sources, as well as one group of children. The adult subjects included 30 migrants and 6 Australian controls who conceived from phase 2 of the pregnancy study (1993–1998; Gulson et al. 1997, 1998). The migrant cohort consisted of 15 pregnant subjects and 15 non-pregnant controls from the former Yugoslavia, former Soviet Union, Poland, Bulgaria, Romania, Albania, and China. A second cohort of pregnant migrants (*n* = 9) were enlisted for phase 3 of the study (1999–2002) in which subjects were supplied with Ca supplements during pregnancy and 6 months postpartum (Gulson et al. 2004). The pregnant subjects were monitored throughout gestation and for 6 months postpartum. In addition, we monitored 10 children of the nonpregnant migrant controls to evaluate the impact of dietary absorption on the Pb burden of adults versus children (Gulson et al. 2001). The ages of the children ranged from 6 to 11 years, and the (nonpregnant) mother–child pairs were monitored from 12 to > 24 months. In summary, there were four groups of subjects: 36 phase 2 adults further stratified into 15 nonpregnant migrant subjects (group 1.NPM) and 21 pregnant migrant and Australian subjects (group 2.P2P), 9 phase 3 migrant adults (group 3.P3P), and 10 migrant children (group 4.MC). None of the subjects was exposed to other potential Pb sources such as deteriorating leaded paints or older Pb-bearing dusts released by renovations and other activities throughout the study period. Geometric mean PbB levels at the time of first blood sampling were < 5 μg/dL (Figure 1).

Informed consent forms (translated into the subjects’ native language) were obtained from each volunteer. This consent form had been reviewed and approved by the Ethics Committee of St. Vincent’s Hospital of Sydney and by the University of Adelaide in Australia. As part of the entry requirements into Australia, all subjects had been declared medically fit.

### Samples and collection

Food sampling involved a 6-day duplicate diet approach to coincide with the quarterly biologic and environmental sampling. Details of the protocols and analytical procedures were described by Gulson et al. (2001). Each daily sampling was blended in a kitchen blender, several portions were taken from each day’s blended diet and composited, and the 6-day composite was then blended in a laboratory blender. Several food samples were analyzed in duplicate to determine the efficiency of homogenization of the blending (Gulson et al. 2001). The diets for the nonpregnant mothers and children were collected (and analyzed) separately.

### Analytical methods

Samples were analyzed by inductively coupled plasma mass spectrometry (ICP-MS) at the Australian Government Analytical Laboratories (Sydney), the laboratories that undertake the Australia New Zealand Food Authority Market Basket Surveys. Samples were measured for Ca, magnesium, sodium, potassium, Ba, Sr, P, Zn, and Cu. Analyses for Fe are available only for pregnant subjects because the study was not focused primarily on micronutrient intake, and the first author failed to notify the laboratory to analyze for Fe in the early part of the study. Approximately 10% of the samples were analyzed in replicate (usually duplicate) for quality control. Pb in blood and food was analyzed by isotope dilution using thermal ionization mass spectrometry. Further details of the Pb methods are given by Gulson et al. (1997).

### Questionnaire

A dietary questionnaire was administered soon after recruitment and repeated at least once at a later date, usually coincident with conception and postpregnancy. Particular attention was directed toward diet, but the questionnaire also covered such aspects as ethnic medication and cosmetics. The questionnaire was supplemented on occasion by inspection of storage areas such as kitchen cupboards and refrigerators to identify the source of any food items that may have been overlooked by the subjects. These approaches were used as an indicator of the types and amounts of food consumption of the subject rather than as a statistical measure.

### Statistical analysis

Notched box plots of untransformed data were produced using MedCalc (MedCalc Software, Mariakerke, Belgium). For other analyses, the dependent variable was PbB (micrograms per deciliter), log_10_ transformed to approximate normality. The independent variables, apart from group and time in months, were Ba (micrograms per day), Ca (milligrams per day), Cu (micrograms per day), Fe (milligrams per day), Mg (milligrams per day), P (milligrams per day), K (milligrams per day), Na (milligrams per day), Sr (micrograms per day), Zn (milligrams per day), and Pb food (micrograms per day). The variables were log_10_ transformed to approximate normality for the purposes of analysis. Although independent variables are not assumed to be normally distributed, normality maximizes the chance of finding relations with the dependent variable (Tabachnick and Fidell 1996).

We used a mixed linear model, as implemented in SPSS (version 13; SPSS Inc., Chicago, IL, USA) for the analyses. The transformed PbB level was the dependent variable, whereas the independent variables were subject (random factor), group (fixed factor, dummy-coded), and time (a numeric variable coded in months, where the time of the first measurement for each subject had a value of zero), along with one or more of the micronutrient measures of interest. Restricted maximum likelihood was used for model fitting, except when making model comparisons, when maximum likelihood was used.

## Results

Some demographic characteristics of the participants along with mean micronutrient values are listed in Table 1. In our previous study reporting progressive results for daily intakes, we found that apart from Ba, there were no significant seasonal differences in daily intake of the elements (Gulson et al. 2001). Significant differences were that the pregnant migrant women (group 2) had higher daily intakes of Ca, K, Mg, Na, Zn, P, and Sr (and the combined variables) than did the nonpregnant migrant women (group 1), and the pregnant Australian women (group 2) had higher daily intakes of Ca, Mg, Zn, P, and Sr (and the combined variables) than did the nonpregnant migrant women (Gulson et al. 2001).

**Figure 2:**
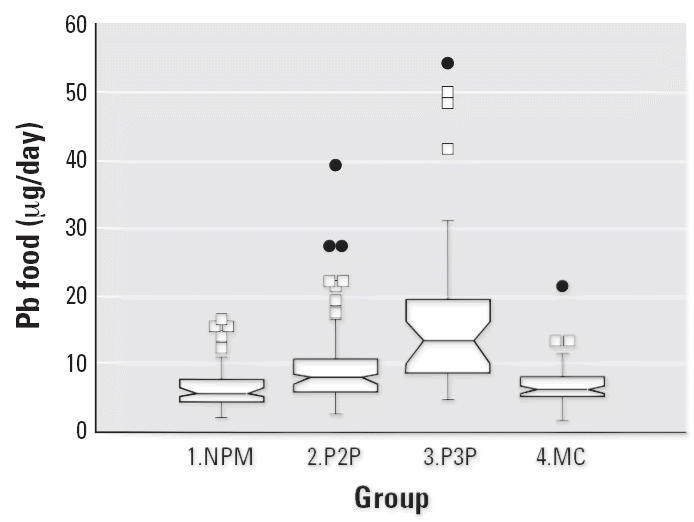
Notched box plot for descriptive statistical data showing daily intakes of Pb from 6-day duplicate diets. There is a significantly higher intake for group 3 (phase 3) migrants.

**Figure 3:**
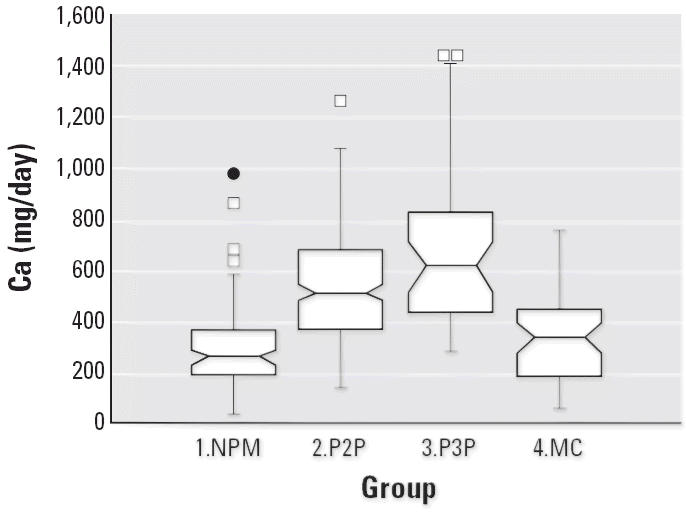
Notched box plot for descriptive statistical data showing daily intakes of Ca from 6-day duplicate diets. The intakes for group 3 (phase 3) subjects are higher than indicated because the Ca supplements were not added to the dietary collections. The recommended daily intake for pregnant subjects is 1,200 mg Ca (National Research Council 1989).

**Figure 4:**
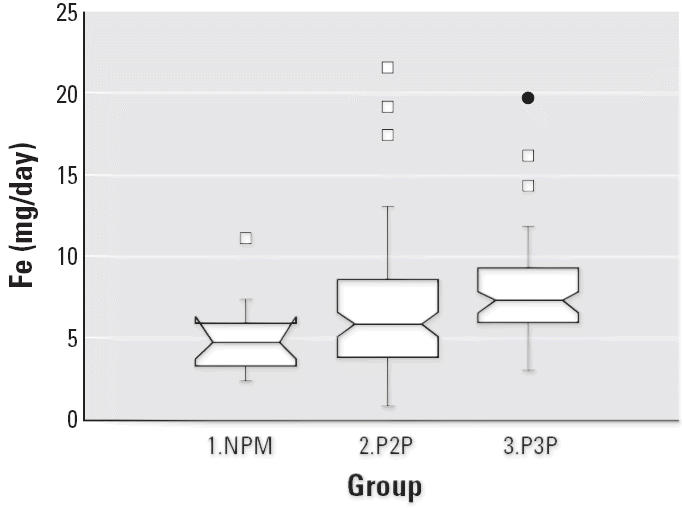
Notched box plot for descriptive statistical data showing daily intakes of Fe from 6-day duplicate diets. The recommended daily intake for such females is 15 mg Fe (National Research Council 1989).

**Figure 5:**
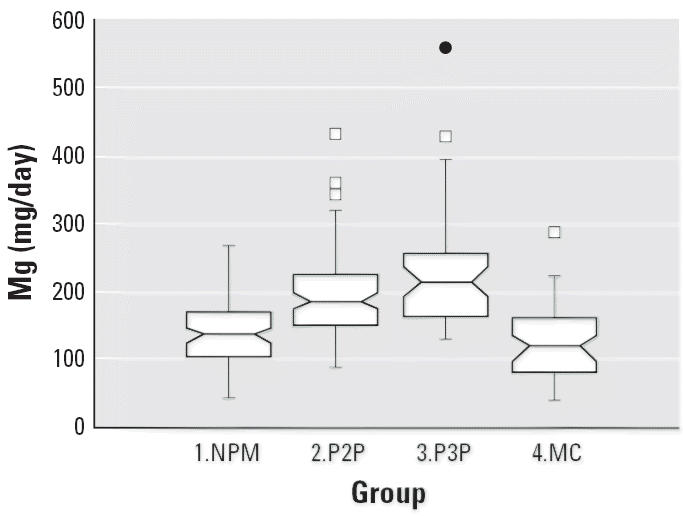
Notched box plot for descriptive statistical data showing daily intakes of Mg from 6-day duplicate diets. The recommended daily intake for such females is 340–355 mg Mg (National Research Council 1989).

**Figure 6:**
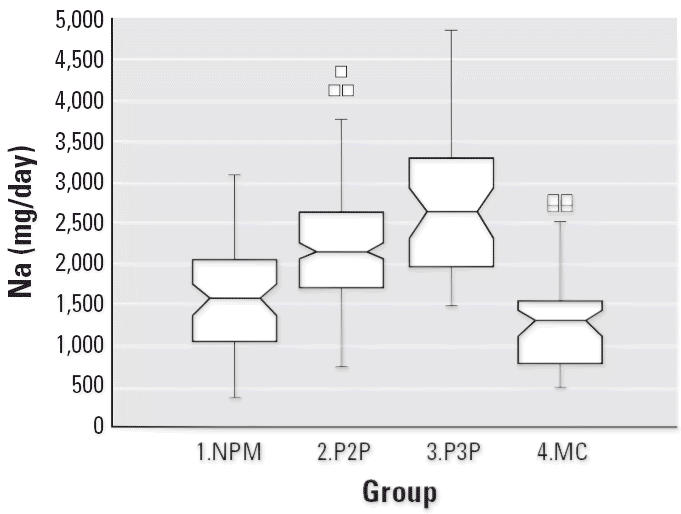
Notched box plot for descriptive statistical data showing daily intakes of Na from 6-day duplicate diets. The U.S. Third National Health and Nutrition Examination Survey (NHANES III) daily intakes for such females are approximately 3,000 mg Na (Alaimo et al. 1994).

**Figure 7:**
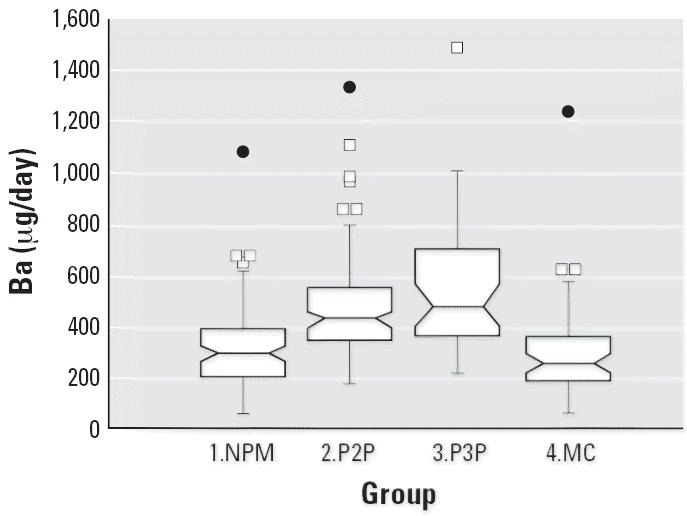
Notched box plot for descriptive statistical data showing daily intakes of Ba from 6-day duplicate diets. No recommended daily intake values are available.

**Figure 8:**
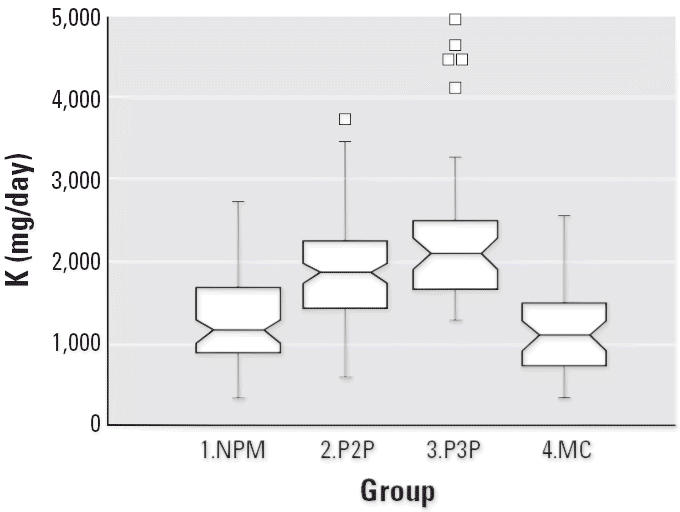
Notched box plot for descriptive statistical data showing daily intakes of K from 6-day duplicate diets. NHANES III daily intakes for such females range from 2,300 to 2,580 mg K (Alaimo et al. 1994).

**Figure 9:**
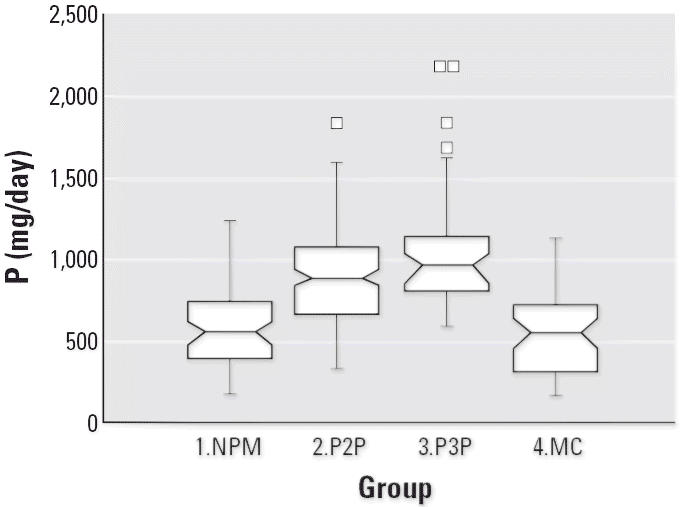
Notched box plot for descriptive statistical data showing daily intakes of P from 6-day duplicate diets. The recommended daily intake for such females is 1,200 mg P (National Research Council 1989).

**Figure 10:**
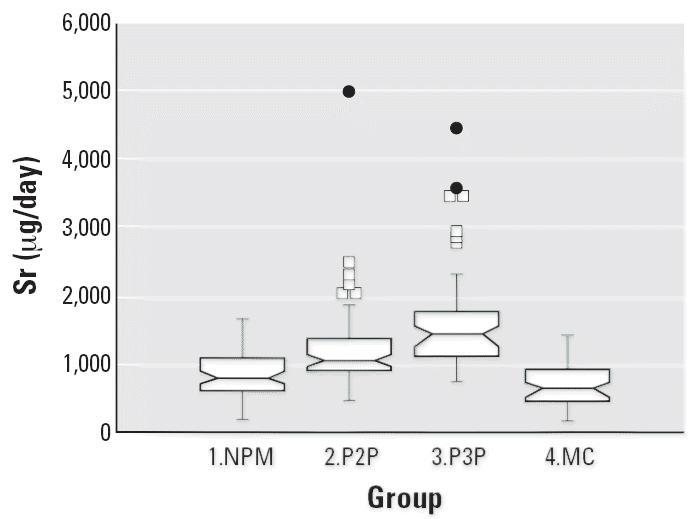
Notched box plot for descriptive statistical data showing daily intakes of Sr from 6-day duplicate diets. No recommended daily intake values are available.

**Figure 11:**
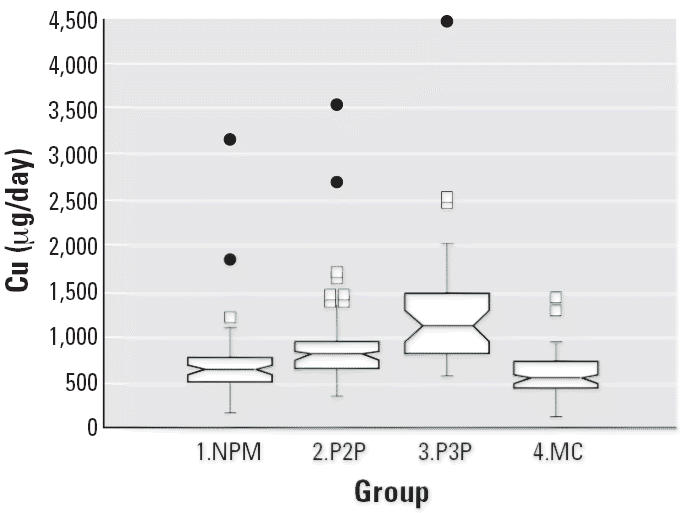
Notched box plot for descriptive statistical data showing daily intakes of Cu from 6-day duplicate diets. No recommended daily intake values are available.

**Figure 12:**
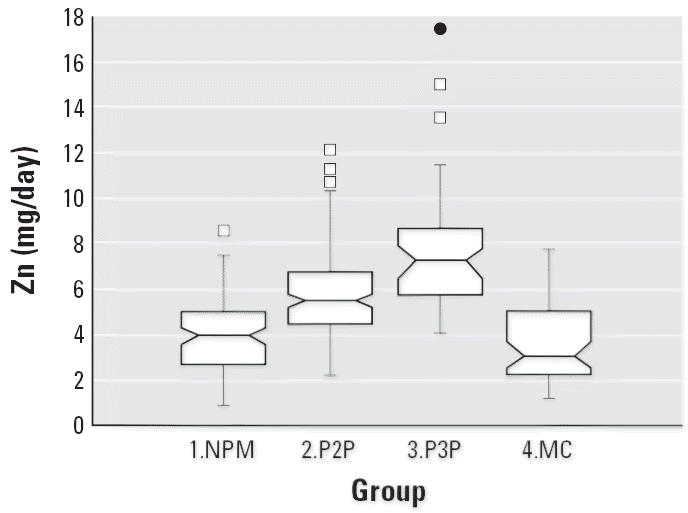
Notched box plot for descriptive statistical data showing daily intakes of Zn from 6-day duplicate diets. The recommended daily intake for such females is 16–19 mg Zn (National Research Council 1989).

**Figure 13:**
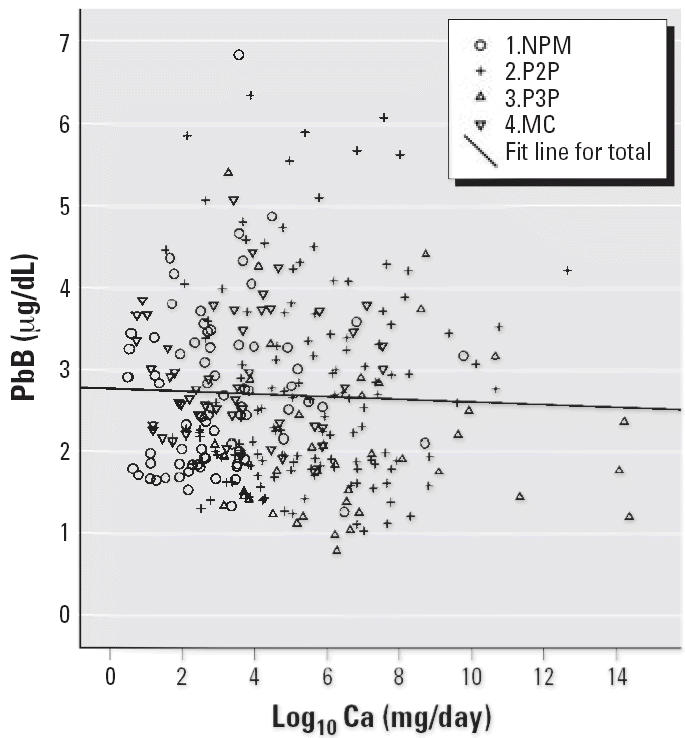
Scatter plot of PbB versus Ca daily intake (log_10_).

Notched box plots for descriptive statistical data for daily intakes of selected micronutrients from 6-day duplicate diets expressed as milligrams per day or micrograms per day are illustrated in Figures 2–12, and a scatter plot of PbB versus daily intake of Ca is shown in Figure 13. The descriptive results for Ca, Mg, Ba, P, Na, and K are similar and suggest that there is no significant difference at the 95% confidence interval between the group 3.P3P and group 2.P2P subjects. In contrast, the daily intakes of Pb, Cu, Zn, and Sr appear to be significantly higher for the group 3.P3P compared with group 2.P2P subjects. Higher daily intakes for Zn and Sr may be partly explained by the amounts of these elements in the Ca supplements because the potential daily intakes from one of the supplements could be approximately 8 mg Zn and for Sr both were approximately 300 mg/day. Supplement contribution is, however, not the explanation for Pb or Cu. The high Pb intakes for some subjects are of concern given that the recommended U.S. daily intake is < 10 μg/day (Bolger et al. 1996).

In the present analysis, the data consisted of 303 observations on four groups of subjects: 36 phase 2 adults further stratified into 15 nonpregnant migrant subjects (group 1.NPM; phase 2 nonpregnant, 75 observations) and 21 pregnant migrant and Australian subjects (group 2.P2P, 139 observations), 9 phase 3 migrant adults (group 3.P3P; 40 observations), and 10 migrant children (group 4.MC; 49 observations). Our previous analysis (Gulson et al. 2001) had shown that there were no significant differences in daily intake between the pregnant migrant and Australian subjects, and the same was true for this sample, so the data for these two groups were combined for the purposes of analysis. The number of observations per individual ranged from 2 to 13. Twenty-seven of the observations for the phase 2 group and one of the observations for the migrant children group were averages of measurements taken on the same day for the same subject. The average time between observations for each subject was a little less than 4 months (overall mean, 3.91), ranging from < 1 month (0.97) to 14 months.

As found in the descriptive data, there is a significant difference (*p* ≤ 0.001) in daily intake of all metals for nonpregnant and pregnant migrant women (Ba, Ca, Cu, K, Na, Mg, Zn, P, Sr, and Pb). However, these differences arise mainly from the group 3.P3P women who are from a different cohort than the group 2.P2P (2) migrant subjects. There is no significant difference between the nonpregnant migrants and the migrant children, but this is partly expected because they resided in the same house and ate much the same diet. The observations of PbB level from the same individuals were highly correlated; the intra-class correlation was 0.68 (95% confidence interval, 0.58–0.79).

A model that included only group and time accounted for 5.9% of the variance of the PbB measurements (calculated with the method of Snijders and Bosker 1999). Neither the effect of time [*B* = −0.00125, *F*(1, 257.2) = 2.72; *p* = 0.100] nor the effect of group [*F*(3, 51.1) = 2.19, *p* = 0.101] was significant. The only pairwise group difference that approached significance was that between the group 3.P3P and group 4.MC subjects, with the latter group having the higher mean [*t*(51.7) = 2.50, *p* = 0.096, Bonferroni adjusted].

When fitted individually with the model containing only time and group, none of the micronutrients was statistically significant. The nearest to a significant relationship with PbB level was for Cu [*B* = −0.0269, *t*(269.2) = 2.84, *p* = 0.093].

A model containing all of the micronutrient variables (except Fe, for which there was a great deal of missing data), along with time and group, accounted for approximately 9.2% of the variance of PbB, an additional 3.5% compared with the model containing only time and group. This increase was not statistically significant [chi-squared(10) = 16.47, *p* = 0.087]. Considering just the variation between subjects in terms of the Pb levels in their blood, the inclusion of the independent micronutrient variables had very little explanatory power: The unexplained variance among subjects in the null model was 0.019, whereas with time and group included the residual subject variance was 0.018, and with all the variables of interest included it was 0.017. This represents a decrease of only around 4.5% from the model including time and group to that including all of the micronutrient variables.

In the model containing all of the micronutrient variables, the coefficients for Cu, Mg, and P were statistically significant (Table 2). However, it is hard to know whether these effects are of substantive significance, given the relatively small sample and the fact that the effect of each variable was tested after adjustment for a large number of correlated variables.

In examining the box plots, group 3.P3P subjects had the highest Pb intakes but the lowest PbB level. Furthermore, this group also has the slightly higher (but not statistically significant different) Ca and higher Zn intakes, two nutrients whose body stores are generally inversely associated with Pb. To test whether the association between Pb intake and PbB may be modified by micronutrient intake, the data were further analyzed using models that included interactions between Pb intake (Pb food) and the other micronutrients. One interaction at a time was tested, adjusted for all other elements to maximize the sensitivity of the tests. In every case, the interaction was negative, indicating that as the level of micronutrient increased, the strength of the relationship between Pb intake and PbB decreased (Table 2). However, the effects were small, and in this relatively small sample, none reached significance except for Cu at a level of about *p* = 0.1.

The extent of the intercorrelation among the measures can be gauged from the fact that a principal-components analysis of the variables of interest (excluding Fe, and adjusting for group and time to exclude correlations due to the effects of these variables) gave rise to a single factor that accounted for 63.8% of the variance of the measures. Each measure was substantially correlated with the factor (0.35–0.94). In a mixed-model analysis with time, group, and the component score as the independent variables, the effect of the factor scores was not significant [*F*(1, 295.3) = 0.03, *p* = 0.863], and the variance accounted for was very similar to that for the model containing only time and group.

## Discussion

The finding that none of the micronutrients is significantly related to PbB levels was surprising and inconsistent with most previous studies, but those studies usually focused on a maximum of four elements, including Ca, Fe, Zn, and Cu or Ca, P, and Mg. In a recent study, however, Schell et al. (2004) found significant inverse relationships of the PbB levels of infants at 6 months age with their intake of Zn, Fe, and Ca, but with Fe only at 12 months of age. Dietary intake was assessed by 24-hr recall at 3 monthly intervals. In a cross-sectional analysis of 747 Boston, Massachusetts, area men 49–93 years of age in the Normative Aging Study, Cheng et al. (1998) found an inverse association between PbB levels and total dietary intake of vitamin C and Fe but not for Ca, P, Zn, or vitamin D.

In an earlier metabolic balance study, Ziegler et al. (1978) observed an inverse relationship between dietary Ca and retention and Pb absorption in young infants. Other studies in humans have also observed an inverse association between PbB levels and Ca intake (Blake and Mann 1983; Heard and Chamberlain 1982; Johnson and Tenuta 1979; Mahaffey et al. 1973, 1986; Sargent et al. 1999; Sorrell et al. 1977). In humans, the Ca–Pb interaction could arise in several ways, including binding of Pb to Ca or its derivatives in the gastrointestinal tract so that it is not available for absorption, competing with Pb in the gastrointestinal tract for transport sites and absorptive mechanisms, and altering the affinity of target tissues for Pb (Barltrop and Khoo 1975). Pb may also interfere with Ca-mediated cellular processes (Dave et al. 1993; Pounds 1984; Pounds et al. 1991).

The presence of other micronutrients besides Ca appears to be an important factor in Pb absorption from the gastrointestinal tract. For example, Pb absorption decreases as Ca (± P) concentrations increase (Blake and Mann 1983; Heard and Chamberlain 1982). Reductions in Pb absorption and retention were noted with both Ca alone (as Ca carbonate) and P alone (as Na phosphate) but Ca was much more effective than P (Blake and Mann 1983; Heard and Chamberlain 1982). Dietary Ca and P were important predictors of blood Pb concentrations for children 12–47 months of age from a low-income population in central Washington, DC (Mahaffey et al. 1976). Likewise, Sorrell et al. (1977) and Johnson and Tenuta (1979) observed inverse correlations between Pb and Ca intake, vitamin D, and milk-based foods. In contrast, we observed no significant association between PbB and dietary Ca or P, although there was a significant association for P (and Mg and Cu) when all micronutrients were included in the model.

Another important factor affecting gastrointestinal absorption is the relative condition of the gut, that is, whether in a fasted or non-fasted state. Radioactive and stable isotope tracer studies have shown that the absence of Ca and other minerals in the gastrointestinal tract at the time of Pb ingestion is a major reason for increased Pb absorption in fasting subjects compared with nonfasting subjects (Blake and Mann 1983; Chamberlain et al. 1978; Heard and Chamberlain 1982; Rabinowitz et al. 1976, 1980). When Ca and other minerals are present, however, differences between fasting and nonfasting subjects are not significant (Heard and Chamberlain 1982; Krebs et al. 1997; Rabinowitz et al. 1980).

Reductions in Pb absorption were also noted in subjects ingesting ^203^Pb in different foods, depending on the Ca, Mg, and P content of the ingested meal (James et al. 1985).

Studies using fasted and nonfasted laboratory animals, including rats, mice, and monkeys, have produced similar result to those in humans (Mahaffey et al. 1973; Meredith et al. 1977; Quarterman et al. 1978). Gastrointestinal absorption of Pb in rats was shown to decrease in the presence of a number of minerals (Barltrop and Khoo 1975), including several analyzed in this study (Na, Ca, K, Mg, Fe, Zn, Cu). In the Barltrop and Khoo (1975) study, low Fe, Cu, and Zn did not increase Pb absorption in rats although their overall low-mineral deficient diet resulted in a 12-fold increase in Pb absorption. They found that increases in Pb absorption due to the lack of the individual minerals containing Ca, P, and Mg did not summate to the 12-fold increase and suggested that the 12-fold increase was caused by a synergistic effect. Using several dietary regimes (high and low fat, protein, minerals, fiber, and vitamins) Barltrop and Khoo (1975) found that only the regime of added minerals decreased Pb absorption. As in the human studies, administration of P without Ca did not produce reductions in Pb retention as great as that for Ca alone or for Ca with P (Barltrop and Khoo 1975).

The length of time over which a study was undertaken may also be an important factor in absorption of Pb from the gastrointestinal tract. Apart from the investigations of Rabinowitz et al. (1976, 1980) of up to 210 days, the other studies involving radioactive tracers were only of short duration of less than 7 days.

A negative association between Zn and Pb has been shown in experimental animal studies to prevent tissue accumulation of Pb by reducing the inhibitory effect of Pb on certain enzymes involved in heme biosynthesis (Dutkiewicz et al. 1979; el-Waseef and Hashim 1985; Flora et al. 1989). Cerklewski (1979) observed beneficial effects of Zn with Pb in pregnant rats, but the postabsorptive interaction was less important than the intestinal interaction of Pb and Zn. However, Barltrop and Khoo (1975) found that low Zn, Fe, Mn, Cu, iodine, and molybdenum did not have any effect on Pb absorption in rats. Results have been mixed for the limited human studies that have addressed the relationship between Zn and Pb (Bárány et al. 2005; Flanagan et al. 1982; Lauwerys et al. 1983; Thomasino et al. 1977). For example, in a study of 85 fasting males and females, Flanagan et al. (1982) observed that Pb retention was not related to body Fe burden or even a 10-fold molar excess of Fe, of Zn, Co, or Ca. In a study of elderly humans, Bunker et al. (1984) found the beneficial effects of Zn to be the reverse of those found in children’s studies.

In animal experiments, Fe deficiency increased the absorption and potential toxicity of Pb (Barton et al. 1978; Hamilton 1978; Ragan 1977; Shukla et al. 1990; Six and Goyer 1970; Wright et al. 1998). Studies in human adults and children have reached similar conclusions (e.g., Bradman et al. 2001; Cheng et al. 1998; Graziano et al. 1990; Hammad et al. 1996; Lanphear et al. 2002; Mahaffey and Annest 1986; Markowitz et al. 1990; Osman et al. 1998; Watson et al. 1980, 1986; Willows and Gray-Donald 2002; Wright et al. 2003; Yip and Dallman 1984; Yip et al. 1981), although there are exceptions such as that observed by Flanagan et al. (1982), described above. In the most recent study, Schell et al. (2004) found lower dietary Fe intakes to be associated with higher PbB levels, at least through the first year of life. On the other hand, in a study of 234 boys and girls at 15 and 17 years of age, Bárány et al. (2005) found the relationships between Fe status and Pb in blood and serum to be equivocal.

There are limited data relating Pb and Mg. Rats fed Pb plus Mg had higher PbB than did rats fed Pb only, and Pb in bone in the Pb–Mg group was lower than the Pb group (Singh et al. 1979). The authors suggested that Mg mobilized Pb from bone with increased amounts of Mg resulted in lower retention and increased excretion of Pb. The individual role of Mg is difficult to evaluate in the rat study of Barltrop and Khoo (1975) because of the complex mineral diet. Soldatovic et al. (1993) found that supplementation with Mg in rabbits effectively reduced the Pb content in blood and enhanced Pb elimination via the urine. In the study of 23 adults ingesting ^203^Pb under fasting and nonfasting (full meal) conditions, James et al. (1985) measured Mg concentrations along with Ca and P, but found it impossible to separate any effects from the individual micronutrients.

To our knowledge, studies of the relationships of PbB and the other micronutrients analyzed in our investigation such as Ba and Sr or even K and Na have not been undertaken in humans, although they are critical in many bodily functions.

There are several limitations to this study. Most of our data are for pregnant adult subjects rather than children, although many studies, especially for Fe deficiency, have involved pregnant animals and humans. In addition, our cohorts comprised subjects from different countries who may have different dietary habits. However, we did not see a country effect in the analyses. Only limited conclusions can be drawn from the Fe data because early samples were not analyzed for Fe. We also have a limited number of subjects and employed a 6-day duplicate protocol, although this is outweighed by the longitudinal sampling for individuals with up to 13 collections. Because this study was focused primarily on pregnancy, we have only limited data for body weights (not presented), so the null findings may be driven by raw intake instead of body weight–adjusted intake. Furthermore, body stores of nutrients (particularly Ca, Zn, and Fe) were not measured, and body stores of these nutrients may be a better predictor of PbB than nutrient intake. It is the body stores that ultimately up- or down-regulate absorption of metals, not the daily intake of metals (Gunshin et al. 1997). There is evidence that multiple divalent metals (including Pb) bind to the Fe transport protein DMT-1 (Gunshin et al. 1997), and Fe deficiency is known to up-regulate this protein (Andrews et al. 1999). This may be why Fe deficiency will up-regulate Pb absorption. Although biomarkers of body stores are correlated with intake, it is nonetheless possible that serum ferritin, bone density, or serum Zn would have predicted PbB, even when daily intake of Fe, Ca, or Zn does not, because these biomarkers are a better measure of long-term intake.

In summary, in our longitudinal sampling of 6-day duplicate diets in pregnant and non-pregnant females and children with low PbB values, we have not found significant relationships between PbB and the daily intake of Ba, Ca, Cu, K, Mg, Na, Zn, P, Sr, and Pb. There was, however, a significant association for P, Mg, and Cu when all micronutrients were included in the statistical analysis, perhaps reflecting a synergistic effect. Unfortunately, these three elements are not commonly analyzed in Pb studies. Although these outcomes would appear to conflict with some other studies in the literature, there is sufficient uncertainty in the literature of Pb–micronutrient relationships, except probably for Fe and Ca, to advocate that supplementation with the micronutrients analyzed here will not benefit adults and children whose Pb exposures are low PbB. However, this study should be followed up with similar human investigations incorporating varying levels of micronutrients in the diet over time.

## Figures and Tables

**Table 1:** Mean PbB, age at time of first sampling, and mean daily intakes for subjects.

Subject	Group and cohort	PbB (μg/dL)	Ba (μg/day)	Ca (mg/day)	Cu (μg/day)	Fe (mg/day)	K (mg/day)	Mg (mg/day)	Na (mg/day)	Zn (mg/day)	P (mg/day)	Sr (μg/day)	Pb food (μg/day)	Average daily weight of food (mg)	Age at first sampling (years)
1001	1.NPM	3.38	319	258	570	NM	1,307	127	1,432	4.36	455	966	8.00	1,000	34
1004	1.NPM	2.91	292	240	654	NM	863	119	1,231	2.61	457	690	5.54	905	33
1009	2.P2P	2.20	430	427	711	4.98	1,635	162	1,822	4.56	758	950	4.62	1,332	21
1013	1.NPM	4.78	391	343	779	4.78	1,861	168	2,682	7.11	719	1,198	6.31	1,455	26
1015	1.NPM	4.34	326	295	747	NM	894	125	729	2.57	412	823	8.27	1,140	37
1016	2.P2P	1.84	556	564	966	11.76	2,472	242	2,838	6.24	920	1,400	9.72	1,741	26
1022	2.P2P	2.46	572	515	1,063	9.26	1,984	219	2,796	8.72	1,020	1,209	9.15	1,681	30
1023	1.NPM	1.76	197	138	494	NM	813	74	871	2.37	272	451	5.24	1,076	33
1025	1.NPM	2.73	265	210	579	3.36	1,060	117	1,382	2.86	430	672	4.92	1,319	28
1029	1.NPM	2.89	634	580	977	NM	2,001	217	1,961	5.03	899	1,299	11.00	1,698	36
1030	1.NPM	1.74	353	263	542	NM	1,095	148	1,936	4.28	603	829	5.20	1,077	19
1031	1.NPM	3.22	128	114	310	NM	653	66	1,226	2.85	323	315	4.10	627	38
1032	1.NPM	2.62	311	320	539	5.21	1,670	160	1,961	5.12	672	1,015	5.68	1,371	24
1035	2.P2P	1.75	358	490	862	6.14	1,464	152	1,910	4.87	790	980	10.39	1,356	33
1041	2.P2P	2.03	430	337	1,088	6.72	1,160	140	1,408	4.06	632	868	5.82	1,303	23
1042	2.P2P	4.41	325	391	696	3.97	1,337	140	2,190	3.95	589	839	5.98	1,463	32
1043	2.P2P	2.07	528	811	854	6.97	2,201	224	2,320	6.64	1,123	1,383	9.49	1,748	32
1045	2.P2P	2.00	390	710	779	1.69	2,232	197	2,976	5.59	1,038	1,078	9.59	1,916	24
1046	1.NPM	1.75	311	436	753	NM	1,485	150	2,689	5.31	839	1,004	7.67	1,663	30
1047	1.NPM	1.89	249	249	754	NM	1,117	122	1,356	3.77	531	639	5.65	1,117	29
1049	2.P2P	4.18	665	669	869	6.85	2,230	265	2,349	6.59	1,095	1,475	9.99	1,684	34
1052	2.P2P	1.95	354	301	668	5.99	1,597	150	2,620	4.68	608	901	9.56	1,337	29
1054	1.NPM	2.67	492	590	879	11.05	2,477	211	2,170	4.63	863	1,118	8.35	1,587	32
1055	2.P2P	2.62	427	534	1,002	5.73	1,847	184	2,462	5.52	825	1,009	10.26	1,657	22
1056	2.P2P	3.17	522	624	843	5.40	2,011	199	2,111	5.55	930	1,727	10.12	1,615	22
1057	2.P2P	4.30	586	582	823	8.07	1,828	197	1,921	6.38	910	1,191	12.23	1,537	25
1064	1.NPM	2.47	192	345	508	2.89	1,125	108	1,393	3.33	516	522	4.24	1,018	28
1065	2.P2P	2.73	393	588	653	5.58	1,723	181	1,309	5.49	903	901	6.81	1,202	22
1066	2.P2P	2.21	333	450	724	5.13	1,272	147	1,569	5.31	728	889	7.44	1,367	32
1069	2.P2P	2.61	354	582	954	4.52	1,767	168	1,832	5.16	782	946	6.73	1,387	34
1084	1.NPM	2.37	397	333	1,394	4.69	1,480	174	2,498	5.60	759	884	8.40	1,159	35
1085	2.P2P	1.69	472	631	805	8.64	2,130	208	2,108	7.58	1,162	1,102	7.46	1,340	36
1090	2.P2P	2.24	197	320	987	7.21	1,368	144	2,380	4.46	539	1,418	13.24	1,559	23
1093	2.P2P	4.39	662	660	1,149	13.06	2,574	278	2,248	8.33	1,202	1,590	13.63	1,561	33
1096	2.P2P	4.24	321	443	549	3.46	917	106	1,292	3.94	515	841	8.16	660	32
1097	2.P2P	2.89	515	314	738	NM	1,332	171	1,831	6.43	584	1,551	15.47	1,252	21
1204	3.P3P	3.19	544	753	1,106	8.66	2,201	224	2,864	7.24	1,011	1,524	10.32	1,722	32
1208	3.P3P	1.60	359	436	741	8.01	1,940	158	2,292	6.86	926	1,064	9.71	1,611	24
1211	3.P3P	2.00	822	951	2,133	12.42	3,211	334	3,614	12.58	1,440	2,638	14.78	3,748	25
1212	3.P3P	2.42	638	755	1,217	6.73	2,357	241	2,616	6.94	969	1,644	12.72	1,862	31
1213	3.P3P	2.22	469	527	925	5.68	1,571	174	2,244	6.81	826	1,164	13.55	1,463	32
1214	3.P3P	1.20	497	530	1,112	6.74	1,994	202	2,419	5.99	854	1,460	33.17	2,654	25
1225	3.P3P	4.82	334	370	953	6.09	1,548	138	2,128	4.76	653	1,161	8.42	1,509	29
1226	3.P3P	1.73	801	1,201	2,135	10.30	4,197	354	4,120	10.89	1,706	3,342	24.75	3,045	20
1229	3.P3P	1.46	350	461	827	5.22	1,699	170	1,667	6.06	735	1,095	17.03	1,716	19
2015	4.MC	2.46	256	219	739	NM	698	97	652	2.17	309	680	7.80	896	7
2023	4.MC	2.31	249	259	485	NM	944	92	909	2.67	380	574	4.93	1,128	8
2029	4.MC	2.58	688	545	917	NM	2,111	217	1,859	5.10	927	1,249	9.16	1,672	11
2031	4.MC	3.50	81	91	318	NM	391	43	773	1.64	209	242	6.04	508	8
2044	4.MC	3.77	238	465	406	NM	1,127	126	969	3.42	601	658	5.44	840	6
2046	4.MC	4.12	334	451	718	NM	1,563	162	2,621	5.75	870	1,082	8.34	1,617	10
2047	4.MC	2.74	254	314	561	NM	945	115	1,123	2.92	571	628	6.00	1,017	6
2052	4.MC	3.04	227	170	466	NM	833	88	1,512	2.24	384	497	6.13	944	6
2054	4.MC	3.66	336	526	549	NM	1,530	145	1,771	3.86	685	807	6.55	1,250	7
2064	4.MC	2.02	259	430	650	NM	1,434	136	1,541	4.36	637	695	6.35	1,032	8

Abbreviations: 1.NPM, group 1, nonpregnant migrant subjects; 2.P2P, group 2, phase 2, pregnant migrant and Australian subjects; 3.P3P, group 3, phase 3, pregnant migrant subjects; 4.MC, group 4, migrant children of group 1 subjects; NM, not measured.

**Table 2:** Results of mixed-model analyses to test interactions between Pb in food and other micronutrients.

Parameter	Estimate	SE	df	*t*-Value	Significance
Intercept	−0.068	1.919	262	−0.035	0.972
Cu (μg/day)	−0.004	0.002	250	−2.259	0.025
Mg (mg/day)	0.104	0.036	258	2.924	0.004
P (mg/day)	−0.020	0.009	269	−2.309	0.022
Ba (μg/day) × Pb food (μg/day)	−0.022	0.045	257	−0.494	0.622
Ca (mg/day) × Pb food (μg/day)	−0.023	0.037	267	−0.630	0.529
Cu (μg/day) × Pb food (μg/day)	−0.025	0.015	259	−1.668	0.097
K (mg/day) × Pb food (μg/day)	−0.009	0.010	262	−0.840	0.402
Mg (mg/day) × Pb food (μg/day)	−0.146	0.113	259	−1.298	0.196
Na (mg/day) × Pb food (μg/day)	−0.009	0.011	255	−0.854	0.394
P (mg/day) × Pb food (μg/day)	−0.005	0.027	266	−0.171	0.864
Sr (μg/day) × Pb food (μg/day)	−0.002	0.011	260	−0.190	0.850
Zn (μg/day) × Pb food (μg/day)	−2.880	3.896	263	−0.739	0.460

df, degrees of freedom.
