# Erratum

**Published:** 2007-03

In Table 1 of the the article by Gulson et al. [ Environ Health Perspect 114:1186–1192 (2006)], the average daily weight of food should have been in grams instead of milligrams.

The authors regret the error.

